# Three-Stage Membrane Treatment of Wastewater from Biodiesel Production-Preliminary Research

**DOI:** 10.3390/membranes12010039

**Published:** 2021-12-28

**Authors:** Magdalena Lech, Agnieszka Klimek, Damian Porzybót, Anna Trusek

**Affiliations:** Department of Micro, Nano and Bioprocess Engineering, Faculty of Chemistry, Wroclaw University of Science and Technology, Wybrzeze Wyspianskiego 27, 50-370 Wroclaw, Poland; Agnieszka.klimek97@gmail.com (A.K.); Damian.porzybot@gmail.com (D.P.); anna.trusek@pwr.edu.pl (A.T.)

**Keywords:** biodiesel wastewater, water reuse, integrated membrane filtration, membrane selection

## Abstract

As biodiesel production as renewable fuel increases, so does the amount of wastewater resulting from this technology. Wastewater is generated during the so-called biodiesel washing, i.e., washing out glycerol and methanol with water. The purified biodiesel must meet international standards, such as EN 14214 or the American ASTM D6751 standard. To fully say that biodiesel technology is environmentally friendly, the amount of wastewater should be minimized. It is also desirable that the purified water can be recycled to the technology. For this purpose, wastewater pre-treated by flotation, during which mainly oils are removed, was subjected to three-stage membrane separation. For each of the stages, the membrane was selected and characterized in terms of its separation capacity and stream stability. Starting with microfiltration, which was mainly aimed at reducing turbidity, affects the permeate flux in the following steps. Then, ultrafiltration and nanofiltration membranes were selected. These membranes were aimed at reducing the concentration of inorganic and organic substances. Consequently the cascade was composed of: MF-0.45 µm, UF-150 kDa, and NF-characterized by an 80% degree of desalination. The final permeate has a salt concentration of less than 0.15 g/L and can be reused in biodiesel technology.

## 1. Introduction

As renewable energy sources are a valued product nowadays, biodiesel as a biofuel derived from chemically processed vegetable oils is gaining value.

An inevitable consequence of expanding the production of biodiesel is the growing amount of generated wastewater. Water is also a very desired product, so deep wastewater treatment to obtain water to recirculate is the aim of modern (clean) technology. The obtained water for reuse must then meet specific requirements depending on the application [1,2].

The first step in biodiesel production is the reaction of the alcohol with the crude oil in the presence of a catalyst to form glycerol and methyl esters. An operation to separate biodiesel and glycerol is then performed, followed by the recovery of alcohol. Recovered alcohol is returned to the reaction, and the methyl ester is directed to the “washing process”, during which a large amount of wastewater is generated [3].

The biodiesel washing operation removes all contaminants and unwanted substances such as unreacted oils, catalysts, excess alcohol, and glycerol. The purified biodiesel must meet international standards such as EN 14214 or the American ASTM D6751 standard. The wet washing of biodiesel has long been the most successful method for removing glycerol and methanol as both are well soluble in water [4].

The amount and properties of biodiesel wastewaters depend on the production system, feedstock, and catalyst. In general, they are highly viscous liquids with an opaque color. The pH range is very high, between 3.3 to 11.2. Its discharging to the public sewerage system would clog the pipes by a significant concentration of oils. It could also adversely affect the activated sludge in biological wastewater treatment plants by inhibiting the growth of microorganisms [3,5,6,7].

Membrane filtration has been used in the industry for many years to treat wastewater [8,9,10]. Membrane filtration, including microfiltration (MF), ultrafiltration (UF), and nanofiltration (NF) is a process group based on a sieving mechanism. The particles retained by them are bigger than 0.1 µm, 5 nm, and 1 nm, respectively, for MF, UF, and NF [11]. After treatment with membrane filtration, wastewater meets the standards that allow discharge into surface waters or return to production. In comparison to conventional approaches, the usage of membranes offers clean, ecological, and energy-efficient solutions. Despite numerous advantages, two phenomena can significantly reduce the permeate flux and thus increase the energy consumption of the process and the operating costs of membrane systems. These phenomena are concentration polarization and membrane fouling, which are closely related, among other things, to the material of the membrane [12,13]. Therefore, membrane choice and process parameters are the first steps in the design of membrane processes.

As wastewater obtained after biodiesel production contains impurities and residues of the oil, it cannot be directly fed to membrane installation. A pre-purification process is needed. Flotation was proposed in the literature, which allows the removal of some major impurities, i.e., large particles and oil [14]. A similar effect was achieved by coagulation, e.g., using the popular salt Fe_2_(SO_4_)_3_ [15].

Despite numerous applications of membrane processes in wastewater treatment, they have not been investigated to treat biodiesel washing wastewater. However, studies can be found in the literature indicating that the planned research can give satisfactory results. Koltuniewicz et al. [16] studied the influence of various factors on decreasing permeate flux during the microfiltration of oily wastewater using a 0.45 µm membrane. Sutrisana et al. [17] also dealt with microfiltration as a technic in oil-wastewater purification. Both of the researcher’s groups proposed microfiltration as a potential method for treating oily wastewater.

Ultrafiltration resulted in 83–100% oils and about 30% COD reduction, while the pH remained unchanged [18]. Nanofiltration is the most popular membrane technique of biodiesel wastewater purification [19,20,21,22]. In this process, removing 85% of COD and decreasing electrical conductivity by about 89% was possible.

Undoubtedly, the nanofiltration process offers the best possibilities for obtaining the process water from biodiesel washing wastewater. However, this is a type of separation in which dense membranes and very high transmembrane pressures are applied. It is reasonable to pre-treat wastewater as much as possible to reduce the scaling and fouling of these sensitive membranes. The application of MF and UF should allow the process water to be obtained at reasonable costs.

The idea of an integrated membrane separation was proposed with success for another kind of wastewater [23,24,25]. Thus, an attempt for biodiesel wastewater treatment by this method has been made. The paper proposes a three-stage cascade of membranes to purify wastewater after washing with biodiesel. The research aimed to configure the membranes for the micro, ultra, and nanofiltration process to obtain water in the permeate of technological water purity. The preliminary studies presented in this paper focused on selecting suitable membranes and their initial characterization in terms of separation capacity for chosen groups of compounds and performance in terms of permeate flux.

## 2. Materials and Methods

### 2.1. Wastewater after Biodiesel Production

Wastewater obtained from the company “Wratislavia Biodiesel SA” was pre-treated by flotation (proprietary method). Waste had come from equipment washing after biodiesel production with base catalyst, thus was slightly alkaline. The main wastewater parameters are presented in Table 1.

### 2.2. Membrane Modules and Installations

The characteristics of the membranes and modules used in this study are presented in Table 2. In different membrane processes, the selectivity of the membrane is defined differently. For MF by the pore diameter size, for UF by the cut-off factor, and for NF, it is expressed by the degree of NaCl removal.

### 2.3. Membrane Installations

A PolyMemTech unit (Warsaw, Poland) and the installation of Millipore (Burlington, VT, USA), were used for the low (MF, UF) and high-pressure (NF) membrane filtration, respectively.

**Figure 1:**
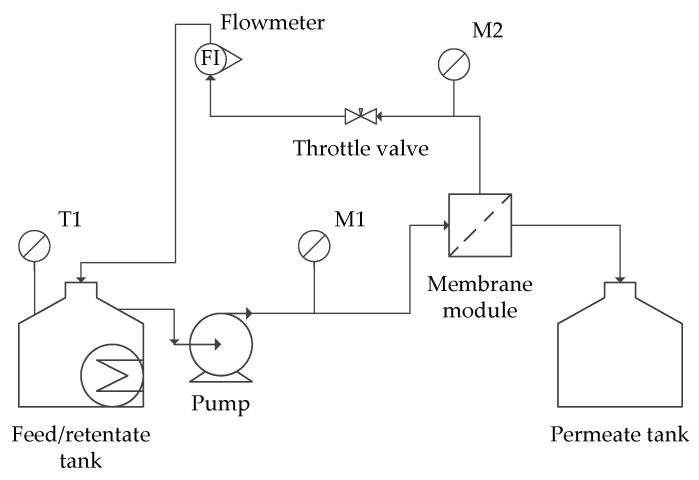
Scheme of the membrane installation. M1, M2—monometers.

The feed solution was dosed using a pressure pump (Zuwa Combistar, 2000-A Hennlich Group, Schärding, Austria). The pressure was kept constant at the values presented in Table 2. The temperature was kept constant (20 °C) by means of an ultrathermostat MA-4 (Julabo, Uster, Switzerland). All separation processes were carried out in one hour. The scheme of installation is presented in Figure 1.

The membranes were regenerated using a protocol:

(1) 2% NaOH heated to 40 °C with NaClO addiction (200 ppm free Cl) for 30 min in close circuit
(2) Distilled water in open circuit
(3) Distilled water in closed circuit for 30 min (pH = 7 of the retentate and permeate)

Then a permeate flux on distilled water was checked. If its value was not as before separation, the protocol was repeated.

### 2.4. Membrane Separations

MF process was carried out under different conditions for two different membranes. During the first separation, the waste was used without any modifications. The second separation utilized the wastewater adjusted to pH = 7. In case of their returning to the production process, it must be neutralized. The last step was to check the effect of the addition of coagulating salt Fe_2_(SO_4_)_3_ at a concentration of 0.5% *w*/*v* [14]. The solution was left for 24 h. After this time, it was separated from the residue by decantation. The clarified solution was subjected to separation.

The degree of membrane retention of the individual components is expressed as commonly expressed in the literature [29,30,31] by the membrane retention (R) calculated as:

(1) $$R\;\left[\%\right]=\left(1-\frac{\mathrm{Permeate}\;\mathrm{parameter}\left(\mathrm{COD},\;\delta ,{\;\mathrm{OD}}_{550}\right)}{\mathrm{Retentate}\;\mathrm{parameter}\left(\mathrm{COD},\;\delta ,{\;\mathrm{OD}}_{550}\right)}\right)\cdot 100\%$$

The feed stream for the ultrafiltration was the permeate obtained from microfiltration on a TAMI membrane with a pore diameter of 0.45 μm without modification of the feed. The aim of this step was the organic (quantified by COD measurement) and inorganic (expressed by conductivity) compounds content partially reduction.

The last stage in the membrane cascade was nanofiltration. The feed of the separation was a permeate obtained from UF separation.

During all separations, the change of the permeate flux was calculated as the ratio of the flux measured at a given time (I(t)) to the initial flux (for a clean membrane—I(0)):

(2) $$\mathrm{Permeate}\;\mathrm{flux}\;\mathrm{drop}=\frac{I\left(t\right)}{I\left(0\right)}$$

### 2.5. Analytical Methods

The permeate flux was measured every 5 min during the process run. Conductivity and pH were measured with electrodes using the multimeter (Crison 5070, Barcelone, Spain). Optical density was measured spectrophotometrically (UV-1800, Shimadzu, Kyoto, Japan) at a wavelength of 550 nm.

The chemical oxygen demand (COD) was determined using ready-made tests by Hanna Instruments HI83224 (Koper, Slovenia). A total of 2 mL of the tested sample was added to 4.25 mL of the ready reagent intended to determine the COD. After thorough mixing, it was incubated for 2 h at 150 °C. Absorbance was measured at 610 nm. The standard curve was prepared with potassium hydrogen phthalate:

(3) $$\mathrm{COD}\;\left[\frac{{\mathrm{mgO}}_{2}}{L}\right]=2.67\cdot 10^{3}\cdot A_{610}$$

All reagents were of analytical grade. They were purchased from Pol-Aura (Roznowo, Poland).

## 3. Results

### 3.1. Microfiltration

This stage of membrane separation aimed to reduce turbidity and clarify the water as effectively as possible.

**Figure 2:**
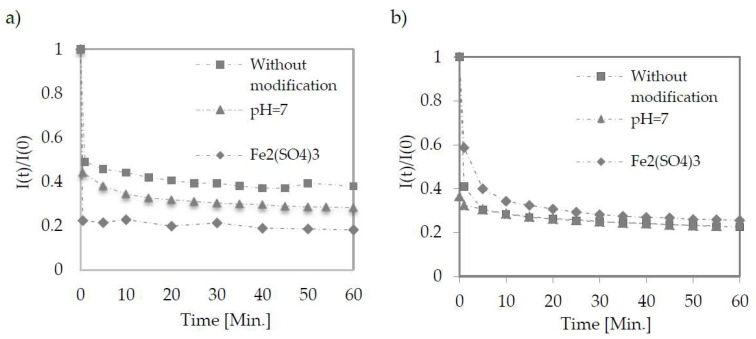
Change in permeate flux during low-pressure filtration using TAMI membranes with pore size of 0.45 μm (**a**) and 0.14 μm (**b**). Points represent the average of five measurements.

A speedy decrease in the permeate flux was observed for each tested membrane and condition (Figure 2). In Table 3, the values of permeate flux in the phase of quasi-static were obtained in each case no later than 5 min after the process.

**Figure 3:**
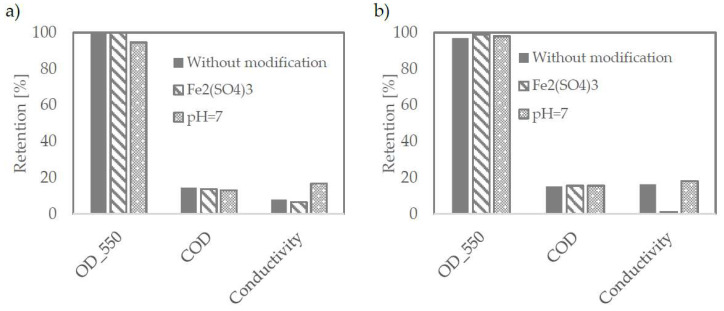
Retention efficiency on TAMI membranes with pore size of 0.45 μm (**a**) and 0.14 μm (**b**).

**Figure 4:**
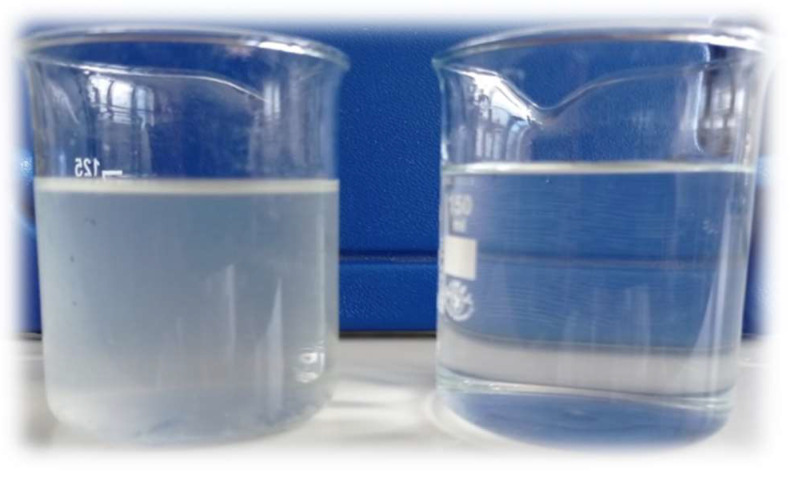
Wastewater before (**a left side**) and after (**a right site**) the microfiltration process on TAMI membrane with pore size of 0.45 mm.

As presented in Figure 3, both tested membranes have proven themselves well at each tested condition. All of them reject particles responsible for turbidity almost totally. Figure 4 shows the wastewater before and after the microfiltration process.

### 3.2. Ultrafiltration

As nearly 100% of suspended compounds were removed in the microfiltration process, the purpose of the subsequent separation steps was to remove organic compounds quantified by COD measurement and inorganic compounds expressed by conductivity.

**Figure 5:**
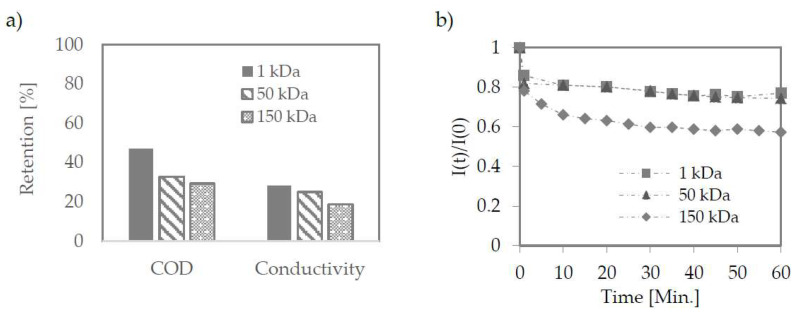
Retention efficiency on ultrafiltration membranes (**a**); Change in permeate flux during ultrafiltration (**b**). The pressure used 2.2, 2.0, 10 bar for 150, 50, 1 kDa membrane.

The membranes were tested at pore size 1–150 kDa. They were prepared with PES (1 kDa) as flat sheets and ceramic tubes (50 and 150 kDa). The retention coefficients for both organic and inorganic matter did not differ significantly despite large differences in the membranes used—Figure 5a.

Despite the most significant permeate flux decrease for a 150 kDa membrane (Figure 5b), in the quasi-static phase, permeate flux density for this membrane was significantly higher compared to the other two membranes (Table 4).

### 3.3. Nanofiltration

**Figure 6:**
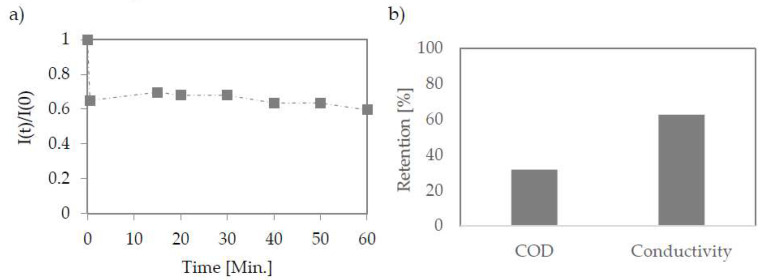
Change in permeate flux during nanofiltration using PA membrane (PCI) (**a**). Retention efficiency on nanofiltration membrane (**b**); The pressure used 15 bar.

The last stage of purification was the use of nanofiltration of water pre-purified by micro-and ultrafiltration. For this type of process, where dense membranes are used, the flux drop is the lowest during the separation, although its value is low app. 0.72 L/(bar·h·m^2^) (Figure 6a).

The qualitative results obtained from the separation showed that the applied membrane is effective to some extent only (Figure 6b). The chemical oxygen demand (COD) reduction was 32%. The high value of this index is due to the high concentration of glycerol. The PCI membrane worked more effectively in the case of salt content reduction. The conductivity was reduced by 63%, and the final salt concentration according to the standard curve made for NaCl was 0.15 g/L.

## 4. Discussion

Wastewater from biodiesel technology is challenging to treat due to the complexity of the matter it contains. It contains both the oily phase and organic and inorganic matter. Just as oils can be fully removed by flotation and microfiltration, the removal of organic matter expressed in the manuscript by COD and inorganic matter defined in conductivity has been brought to the required standards.

The final COD value met Polish environmental protection (Dz. U. z 2015 r. poz.139) standards, as it was lower than 1000 mgO_2_/L. The salt concentration in the final permeate was 0.15 g/L. Based on the Polish regulations (collective sewage disposal rules Dz. U. z 2015 r. poz.139), the salt content should be lower than 1 g/L to meet the standards for wastewater directed to the urban rivers. However, since the obtained permeate also meets process water requirements, it should be recycled to the technology, which will allow for a significant reduction in water consumption.

Comparing the obtained results with those available in the literature is quite difficult. The tested wastewater is quite specific. After the oil phase separation process (by flotation), it already contains a small amount of oil. On the other hand, it contains quite large amounts of salt and glycerin. The first mainly comes from saponification reactions and feedstock, which are usually used in cooking oils. The second component, glycerin, is the second product of transesterification, i.e., biodiesel production.

In the literature on the subject, single-stage membrane separation processes were used. Firmam et al. [20] tested an ultrafiltration membrane made of polyvinylidene polyfluoride/polyvinylpyrrolidone with a mean pore diameter value of ~19 nm. It was concluded that the studied membrane retains between 83–100% of oils and fats, and between 20–40% of COD, whereas salts are not rejected. Better results, especially with regard to the retention of inorganic substances were obtained by Mozaffarikhaha & Kargari [21] using the NE-90 nanofiltration membrane.

In one of the most recent studies (April 2021), J. Torres et al. [18] tested the treatment of (simulated) wastewater from biodiesel using nanofiltration membranes. A polymer membrane (selective layer-polyamide) with a cut-off point of 200 Da and a ceramic membrane of 450 Da, respectively, were tested. COD and conductivity were measured. In both cases, the permeate meeting the required standards were not obtained. The paper ends with the conclusion that the nanofiltration process should be coupled with some other method. Our research confirmed this, where success was achieved in the cascade of membrane processes.

**Figure 7:**
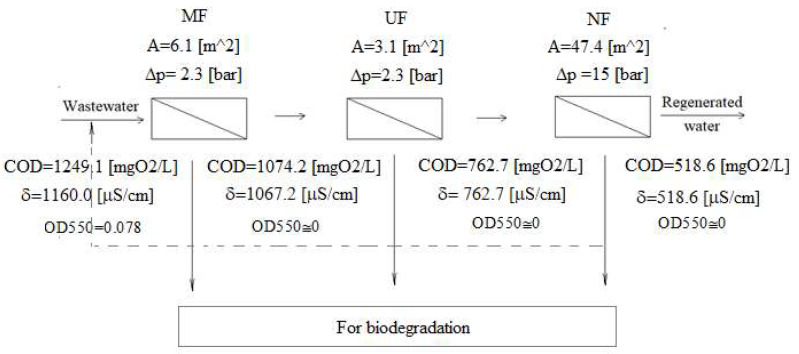
Project of a three-stage membrane installation. At each stage, the permeate flux to the retentate flux is 4:1.

Despite the multistage treatment process, a significant decrease in permeation fluxes was observed at each membrane separation process. However, these streams stabilized quickly, and hence, the stable operation of the installation can be assumed. Figure 7 shows a preliminary design for a three-stage membrane installation. It was assumed that at each stage, the permeate flux (controlled by the size of the area and the degree of retentate recirculation) is four times higher than the retentate flux. The pump must be selected for each process in terms of the required pressure (MF-UF-NF increasing in series) and flux (NF-UF-MF increasing). Hence, the NF process pump is the most expensive investment and operation. Still, thanks to the cascade of preceding processes (MF-UF), the nanofiltration process is carried out on a smaller stream (reduced by MF and UF retentate compared to the original one). The proposed values of parameters should be verified on an industrial installation.

From the above analysis, it can also be seen that the first stage is primarily responsible for clarifying the wastewater. As a result of the MF separation, mainly solid particles (from the raw material used for the production of biodiesel) and oil residues unseparated in the flotation step are retained. The second stage already partially retains organic compounds (mainly oil microdroplets not retained by MF), reducing the COD value slightly. We observe an unexpectedly high degree of conductivity decrease. The probable reason is removing ions existing in a complex with organic/colloidal materials [32]. The last step—nanofiltration decreases COD and conductivity, which is predictable due to partially dense membranes with possibly very small pores. It is suspected that without the presence of glycerin (which improves the dense membrane permeability [33]), the retention of compounds responsible for both parameters would be improved.

Retentates from each separation stage (also matter collected as the so-called filter cake) can undergo biodegradation. Strains capable of having been described in the literature are described [34,35,36]. A membrane bioreactor is particularly recommended for this purpose [37,38].

## 5. Conclusions

The paper describes the attempt to purify the wastewater generated after the biodiesel washing process was made. The wastewater used for the tests was initially purified from the oils by flotation.

The first membrane separation was microfiltration, where membranes with two different pore diameters—0.14 and 0.45 µm—were tested. At this stage, it was additionally investigated how the waste modification by adjusting the pH to 7, and the additional step of pretreatment by coagulation and later sedimentation, influence efficiency. The most satisfactory results (full retention of solids) were obtained by using a 0.45 µm membrane and separating the wastewater immediately after flotation. The permeate stream from microfiltration was directed on the ultrafiltration module.

During ultrafiltration, the stream has been partially purified of both dissolved organic and inorganic matter. Due to the very high dependence of permeate flux on pore size, the membrane with the highest tested cut-off of 150 kDa was chosen. The permeate stream from ultrafiltration was directed on the nanofiltration module.

Using nanofiltration, it was possible to obtain water with the parameters of the process water (salt content in the final permeate was below 0.15 g/L.

The proposed configuration (MF-UF-NF) makes it possible to obtain water that can be returned to biodiesel purification. At the same time, it provides the most significant fluxes of permeates. However, the conducted research is only a guideline for the design of an industrial installation. The selection of membrane surfaces, appropriate pressures, and the development of a self-cleaning membrane procedure during separation must be performed under real (industrial) conditions.

The paper proposes that the obtained by-products of treatment—retentates—may be biodegraded. They contain relatively little oil (most of which has been removed in the flotation process). Still, they already contain a lot of organics (including organic carbon sources such as glycerin) and inorganic, so they should be readily biodegradable. However, it should be more deeply studied to call the process entirely environmentally friendly.

## Figures and Tables

**Table 1:** Characteristic of wastewater used in the experiments.

pH	Conductivity [mS/cm]	OD550	COD [mgO_2_/L]	Color
**8**	1.45 ± 0.11	0.110 ± 0.09	1249 ± 86	White (opal)

**Table 2:** The parameters of the membrane modules used during research.

	Producer	Selectivity	Contact Angle [°]	Membrane Material	Module Type	Area [m^2^]	Water Flux [L/(h*m^2^)] (Applied Pressure [bar])
MF (Tami 0.45 µm)	Tami Industries, Nyons, France	0.45 µm	50 [26]	ZrO_2_/TiO_2_	Tubular 7-channel	0.0130	708.2 (2.3)
MF (Tami 0.14 µm)		0.14 µm		ZrO_2_/TiO_2_	Tubular 7-channel	0.0130	266.3 (2.1)
UF (Tami 150 kDa)		150 kDa	42 [26]	ZrO_2_/TiO_2_	Tubular 7-channel	0.0130	222.0 (2.2)
UF (Tami 50 kDa)		50 kDa		ZrO_2_/TiO_2_	Tubular 1-channel	0.0045	150.0 (2.0)
UF (PolyMemeTech 1 kDa)	PolyMemTech, Warsaw, Poland	1 kDa	60 [27]	PES	Flat	0.0143	80.7 (10.0)
NF	PCI, Hampshire, UK	80% DE	91 [28]	PA	Tubular 2-channel	0.0236	105.0 (15.0)

**Table 3:** Permeate flux in the phase of quasi-static during the microfiltration process. The process was carried out at the pressure 2.1–2.3 bar.

	TAMI 0.45 μm	TAMI 0.14 μm
Without modification	56.96 L/(bar·h·m^2^)	33.33 L/(bar·h·m^2^)
After waste pH correction	92.17 L/(bar·h·m^2^)	39.52 L/(bar·h·m^2^)
After coagulation with Fe_2_(SO_4_)_3_,	117.83 L/(bar·h·m^2^)	34.29 L/(bar·h·m^2^)

**Table 4:** Permeate flux in the phase of quasi-static during the ultrafiltration process. The process was carried out at pressure 2.2, 2.0, 10 bar for 150, 50, 1 kDa membrane.

TAMI 150 kDa	TAMI 50 kDa	PolyMemTech 1 kDa
88.64 L/(bar·h·m^2^)	40.11 L/(bar·h·m^2^)	1.03 L/(bar·h·m^2^)

## Data Availability

Not applicable.

## References

[1] Anderson J. (2003). The Environmental Benefits of Water Recycling and Reuse. Water Sci. Technol. Water Supply.

[2] De Gisi S., Galasso M., De Feo G. (2013). Full-Scale Treatment of Wastewater from a Biodiesel Fuel Production Plant with Alkali-Catalyzed Transesterification. Environ. Technol..

[3] Daud N.M., Abdullah S.R.S., Hasan H.A., Yaakob Z. (2015). Production of Biodiesel and Its Wastewater Treatment Technologies. Proc. Safety Environ. Prot..

[4] Osarumwense J.O., Audu T.O.K., Aluyor E.O., Akhabue C.E. (2021). Effect of Process Variables on Volumetric Mass Transfer Coefficient in Wet Purification of Crude Biodiesel. Biofuels.

[5] Cheerawit R., Sawain A., Suksaroj T., Suksaroj C. (2011). Enhanced Efficiency of Dissolved Air Flotation for Biodiesel Wastewater Treatment by Acidification and Coagulation Processes. Desalination.

[6] Ichiro S.K., Kawamoto Y., Fujii E., Kohda J., Nakano Y., Yano T. (2005). Biological Treatment of Wastewater Discharged from Biodiesel Fuel Production Plant with Alkali-Catalyzed Transesterification. J. Biosci. Bioeng..

[7] Veljković V.B., Stamenković O.S., Tasić M.B. (2014). The Wastewater Treatment in the Biodiesel Production with Alkali-Catalyzed Transesterification. Renew. Sustain. Energy Rev..

[8] Ezugbe E.O., Rathilal S. (2020). Membrane technologies in wastewater treatment: A review. Membranes.

[9] Goh P.S., Ismail A.F., Ng B.C., Abdullah M.S. (2019). Recent progresses of forward osmosis membranes formulation and design for wastewater treatment. Water.

[10] Awad E.S., Sabirova T.M., Tretyakova N.A., Alsalhy Q.F., Figoli A., Issam K. (2021). A mini-review of enhancing ultrafiltration membranes (UF) for wastewater treatment: Performance and stability. Chem. Eng..

[11] Rautenbach R. (1996). Procesy Membranowe: Podstawy Projektowania Modułów i Instalacji.

[12] Meng S., Zhang M., Yao M., Qiu Z., Hong Y., Lan W., Xia H., Jin X. (2019). Membrane fouling and performance of flat ceramic membranes in the application of drinking water purification. Water.

[13] Ma C., Yi C., Li F., Shen C., Wang Z., Sand W., Liu Y. (2020). Mitigation of membrane fouling using an electroactive polyethersulfone membrane. Membranes.

[14] Shuang L.S., Yang C., Pan Y., Zhang J.H. (2015). Preparation of Aluminum-Ferric-Magnesium Polysilicate and Its Application on Oily Sludge. J. Serbian Chem. Soc..

[15] Li X.B., Liu J.T., Wang Y.T., Wang C.Y., Zhou X.H. (2007). Separation of Oil from Wastewater by Column Flotation. J. China Univ. Min. Technol..

[16] Koltuniewicz A.B., Field R.W., Arnot T.C. (1995). Cross-Flow and Dead-End Microfiltration of Oily-Water Emulsion. Part I: Experimental Study and Analysis of Flux Decline. J. Membr Sci..

[17] Sutrisna P.D., Candrawan J., Tangguh W.W. (2019). Microfiltration of Oily Waste Water: A Study of Flux Decline and Feed Types. IOP Conf. Ser. Mater. Sci. Eng.

[18] Torres J.J., Cuello M., Ochoa N.A., Pagliero C. (2021). Biodiesel Wastewater Treatment Using Nanofiltration Membranes. Proc. Safety Environ. Protect..

[19] Ali H., Mohammad A.W., Ahmad A. (2017). The Effect of Wastewater Pretreatment on Nanofiltration Membrane Performance. J. Water Reuse Desalin..

[20] Firman L.R., Ochoa N.A., Marchese J., Pagliero C.L. (2018). Treatment of Aqueous Effluents from the Biodiesel Industry Using Membrane Technology. Rev. Matera.

[21] Kosar M., Ali Kargari A. (2017). Treatment of Biodiesel Production Wastewater by a Commercial Nanofiltration System. Desalin. Water Treat..

[22] Torres J.J., Arana J.T., Ochoa N.A., Marchese J., Pagliero C. (2018). Biodiesel Purification Using Polymeric Nanofiltration Composite Membranes Highly Resistant to Harsh Conditions. Chem. Eng. Technol..

[23] Lech M., Trusek A. (2021). Whey management based on bioreactor and membrane processes: Clean technology gaining valuable components of whey. Desalin. Water Treatm..

[24] Rosenwinkel K.H., Wagner J., Nagy J. (2000). Membrane methods for industrial wastewater treatment. Chem. Ing. Tech..

[25] Marcucci M., Ciabatti I., Matteucci A., Vernaglione G. (2003). Membrane technologies applied to textile wastewater treatment. Ann. N. Y. Acad. Sci..

[26] Ha C. (2013). Ozonation and/or Coagulation-Ceramic Membrane Hybrid for Filtration of Impaired-Quality Source Waters. Ph.D. Thesis.

[27] Cheryan M. (1998). Ultrafiltration and Microfiltration Handbook.

[28] Pang R., Zhang K. (2015). High-flux polyamide reverse osmosis membranes by surface grafting 4-(2-hydroxyethyl)morpholine. RSC Adv..

[29] Sikorska W., Wasyleczko M., Przytulska M., Wojciechowski C., Rokicki G., Chwojnowski A. (2021). Chemical Degradation of PSF-PUR Blend Hollow Fiber Membranes—Assessment of Changes in Properties and Morphology after Hydrolysis. Membranes.

[30] Hadi S., Mohammed A., Jubouri S., Abd M.F., Majdi H.S., Alsalhy Q.F., Rashid K.T., Ibrahim S.S., Issam K., Figoli A. (2020). Experimental and theoretical analysis of lead Pb(2+)and Cd(2+) retention from a single salt using a hollow fiber PES membrane. Membranes.

[31] Niestroj-Pahl R., Stelmaszyk L., ElSherbiny I.M.A., Abuelgasim H., Krug M., Staaks C., Birkholz G., Horn H., Li T., Dong B. (2020). Performance of layer-by-layer-modified multibore (R) ultrafiltration capillary membranes for salt retention and removal of antibiotic resistance genes. Membranes.

[32] Kovalchuk N.M., Dunn J., Davies J., Simmons M.J.H. (2019). Superspreading on Hydrophobic Substrates: Effect of Glycerol Additive. Colloids Interfaces.

[33] https://www.ultrapurewater.com/articles/pharma/practical-examples-of-uf-use-in-pharmaceutical-compendial-water-systems.

[34] de Souza M.M., Colla T.S., Bucker F., Ferrao M.F., Huang C.T., Andreazza R., de Oliveira Camargo F.A., Bento F.M. (2016). Biodegradation potential of Serratiamarcescens for diesel/biodiesel blends. Intern. Biodeter. Biodegradat..

[35] Wu S., Yassine M., Suidan M.T., Venosa A.D. (2015). Anaerobic biodegradation of soybean biodiesel and diesel blends under methanogenic conditions. Water Res..

[36] Ye C., Ching T.H., Yoza B.A., Masutani S., Li Q.X. (2017). Cometabolic degradation of blended biodiesel by Moniliella wahieum Y12(T) and Byssochlamys nivea. Intern. Biodeter. Biodegradat..

[37] Trusek-Holownia A. (2008). Wastewater treatment in a microbial membrane bioreactor—A model of the process. Desalination.

[38] Giacobbo A., Feron G.L., Siqueira Rodrigues M.A., Bernardes M.A., Meneguzzi A. (2011). Use membrane bioreactor for wastewater treatment. Holos.

